# Polycystic ovary syndrome (PCOS) and COVID-19: an overlooked female patient population at potentially higher risk during the COVID-19 pandemic

**DOI:** 10.1186/s12916-020-01697-5

**Published:** 2020-07-15

**Authors:** Ioannis Kyrou, Emmanouil Karteris, Tim Robbins, Kamaljit Chatha, Fotios Drenos, Harpal S. Randeva

**Affiliations:** 1 Warwickshire Institute for the Study of Diabetes, Endocrinology and Metabolism (WISDEM), University Hospitals Coventry and Warwickshire NHS Trust, Coventry, CV2 2DX UK; 2 Aston Medical Research Institute, Aston Medical School, Aston University, Birmingham, B4 7ET UK; 3 Warwick Medical School, University of Warwick, Coventry, CV4 7AL UK; 4 College of Health and Life Sciences, Brunel University London, Uxbridge, UB8 3PH UK; 5 Institute of Digital Healthcare, WMG, University of Warwick, Coventry, CV4 7AL UK; 6 Department of Biochemistry and Immunology, University Hospitals Coventry and Warwickshire NHS Trust, Coventry, CV2 2DX UK; 7 Institute of Cardiovascular Science, University College London, London, UK

**Keywords:** Polycystic ovary syndrome, PCOS, COVID-19, Severe acute respiratory syndrome coronavirus-2, SARS-CoV-2, Diabetes, Obesity, Hypertension, Androgens, Vitamin D

## Abstract

**Background:**

In women of reproductive age, polycystic ovary syndrome (PCOS) constitutes the most frequent endocrine disorder. Women with PCOS are considered to typically belong to an age and sex group which is at lower risk for severe COVID-19.

**Main body:**

Emerging data link the risk of severe COVID-19 with certain factors such as hyper-inflammation, ethnicity predisposition, low vitamin D levels, and hyperandrogenism, all of which have known direct associations with PCOS. Moreover, in this common female patient population, there is markedly high prevalence of multiple cardio-metabolic conditions, such as type 2 diabetes, obesity, and hypertension, which may significantly increase the risk for adverse COVID-19-related outcomes. This strong overlap of risk factors for both worse PCOS cardio-metabolic manifestations and severe COVID-19 should be highlighted for the clinical practice, particularly since women with PCOS often receive fragmented care from multiple healthcare services. Comprehensively informing women with PCOS regarding the potential risks from COVID-19 and how this may affect their management is also essential.

**Conclusion:**

Despite the immense challenges posed by the COVID-19 outbreak to the healthcare systems in affected countries, attention should be directed to maintain a high standard of care for complex patients such as many women with PCOS and provide relevant practical recommendations for optimal management in the setting of this fast moving pandemic.

## Background

The global outbreak of the new disease (COVID-19) caused by the novel severe acute respiratory syndrome (SARS) coronavirus-2 (SARS-CoV-2) has reached pandemic status in March 2020 [1, 2]. Although asymptomatic or mild in most cases, COVID-19 may cause severe illness with increased mortality in high-risk patients [3]. This has forced governments in affected countries to impose various degrees of quarantine and self-isolation measures in order to reduce the COVID-19 incidence and mortality [4]. An increasing body of clinical evidence indicates that the incidence of severe COVID-19 is significantly higher in older vs. younger adults and in men vs. women [5–12]. Certain pre-existing comorbidities, including chronic cardio-metabolic diseases such as diabetes, obesity, and hypertension, have also been widely recognized as key risk factors for severe COVID-19 [11, 13–17].

In women of reproductive age, polycystic ovary syndrome (PCOS) constitutes the most frequent endocrine disorder with a prevalence which may reach or even exceed 10–15%, depending on the studied population and the applied diagnostic criteria [18–20]. Following exclusion of related disorders (e.g., hyperprolactinemia, hypothyroidism, and non-classic congenital adrenal hyperplasia), both ovarian dysfunction (i.e., chronic oligo- or anovulation) and hyperandrogenism (clinical, i.e., hirsutism, and/or biochemical, i.e., increased free testosterone or free androgen index) are the key features in order to establish the diagnosis of PCOS [18–20]. Polycystic ovary morphology on ultrasound has also been introduced as another potential feature for diagnosing PCOS according to the Rotterdam PCOS diagnostic criteria [18–20]. Of note, despite the high prevalence of PCOS, its underlying etiology and pathophysiology are still not fully clarified, while PCOS management in routine clinical practice remains fragmented (e.g., between general practitioners, endocrinologists, and gynecologists), and with significant knowledge gaps among physicians regarding the diagnosis, treatment, and breadth of PCOS features [19–22]. As such, it is not uncommon for women with PCOS to fall between the gaps of relevant healthcare services even in the absence of the extreme pressure applied to clinical practice by the COVID-19 pandemic.

## Overlap between common PCOS features and identified risk factors for severe COVID-19

### Cardio-metabolic comorbidity

**Fig. 1:**
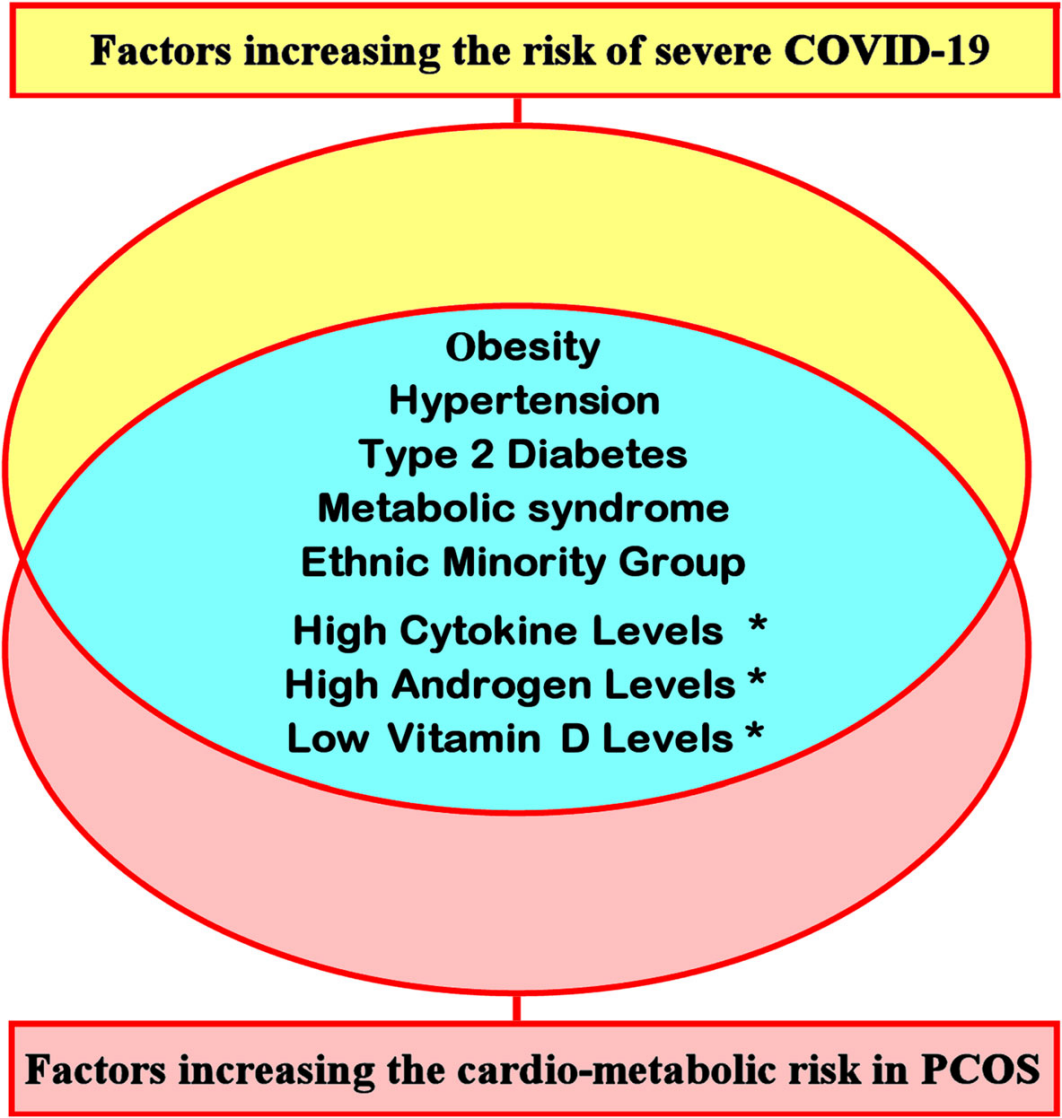
Overlap between common factors which promote an unfavorable cardio-metabolic profile in women with polycystic ovary syndrome (PCOS) and factors which, based on the existing data, appear to increase the risk for severe COVID-19 (*further research is required to clarify the potential links between adverse COVID-19-related outcomes and hyper-cytokinemia, hyperandrogenemia, and vitamin D deficiency)

PCOS is a complex and partly heterogeneous endocrine disorder, often exhibiting a close association with obesity, insulin resistance, type 2 diabetes (T2DM), hypertension, dyslipidemia, obstructive sleep apnea (OSA), and non-alcoholic fatty liver disease (NAFLD) [19, 23–25]. Indeed, depending on the utilized PCOS definition and the demographics of studied populations, up to 75% of women with PCOS also have obesity, with increased central adiposity even independently of the underlying body mass index (BMI) [19, 23]. This common PCOS phenotype is linked to heightened insulin resistance and hyperandrogenism; thus, women with PCOS exhibit markedly increased prevalence of impaired glucose tolerance (IGT), T2DM, and metabolic syndrome [19, 23, 24]. Overall, meta-analysis data suggest a 4-fold higher T2DM prevalence among women with PCOS which is both additive and independent to obesity [26, 27]. As such, it is clear that cardio-metabolic diseases which are common in women with PCOS [19, 23, 24] exhibit significant overlap with the risk factors predisposing to severe COVID-19 (Fig. 1) [11, 13–17]. This overlap between the adverse cardio-metabolic profile of women with PCOS and key identified risk factors for worse clinical outcomes of COVID-19 suggests that this common female patient population is potentially at higher than expected risk if confronted with a SARS-CoV-2 infection. Notably, in the context of the fragmented management of PCOS, superimposed comorbidities such as T2DM often “overshadow” the underlying PCOS diagnosis in affected women; hence, the latter should not be neglected when considering the comorbidities in female COVID-19 patients, so that accurate characterization of the pre-existing risk profile of these women is documented in relevant records/databases.

### Hyperandrogenism

Compelling evidence indicates that, compared to women, men exhibit higher predisposition to developing severe COVID-19, independent of age [8, 12]. The molecular mechanisms which may facilitate this male predisposition for severe COVID-19 are currently investigated, with particular focus placed on the role of angiotensin-converting enzyme 2 (ACE-2) which is activated by the SARS-CoV-2 spike proteins and acts as one of the key mediators for SARS-CoV-2 entry into host cells [8, 28–30]. As such, attention is drawn on the potential effects of sex hormones on ACE-2 expression, with animal data indicating that ACE-2 expression and activity is influenced by sex hormones in various tissues, such as the myocardium, adipose tissue, and kidneys [8]. Interestingly, a preliminary observation has been recently published regarding the high frequency of male pattern hair loss in hospitalized COVID-19 men, further suggesting that androgens might be implicated in COVID-19 severity [31]. Indeed, prompted by the evidence that male sex predisposes to severe COVID-19 and by the fact that male sex hormones impact on the ACE-2 pathway which facilitates the entry of SARS-CoV-2 into host cells [8, 28–30], Goren et al. documented that significant androgenic alopecia was a common feature (71%) in a small cohort of hospitalized male COVID-19 patients [*n*, 41; mean age (range), 58 (23–79) years] [31]. Accordingly, a potential association between androgens and COVID-19 severity appears plausible and could further support the hypothesis that anti-androgens might represent an additional potential intervention against severe COVID-19 [31–34]. This hypothesis also becomes relevant in the context of PCOS, since women with PCOS on one hand exhibit hyperandrogenism (e.g., androgenic alopecia), while on the other hand may already be under treatment with anti-androgens (e.g., spironolactone or finasteride) [19, 20, 35]. At the moment, there is a paucity of data regarding anti-androgen therapy in women with PCOS in the context of COVID-19; hence, this is an additional aspect of the PCOS management which should be studied during this pandemic. Of note, a male PCOS equivalent syndrome has been proposed, which is characterized by early-onset androgenic alopecia in combination with one or more manifestations such as worse gonadal steroidogenesis, decreased sex hormone binding globulin (SHBG), and increased dehydroepiandrosterone sulphate (DHEAS) circulating levels, as well as insulin resistance, T2DM, obesity, and hypertension [36, 37]. As such, there is a potential analogy between women with PCOS and a male PCOS equivalent syndrome that are both linked to coexisting comorbidities (e.g., T2DM, obesity, and hypertension) which, as aforementioned, predispose to severe COVID-19.

However, it should be also noted that at the time of writing, there is a pre-print publication of a retrospective study from Germany which reports that severe testosterone and dihydrotestosterone deficiencies were noted in a small cohort of critically ill COVID-19 men [*n*, 35; median age (range), 62 (31–80) years] [38]. Moreover, in the small number of female COVID-19 patients of this study [*n*, 10; median age (range), 67.5 (54–84) years], a positive correlation was noted between testosterone levels and pro-inflammatory cytokines (e.g., IL-6) [38]. As the publications, including pre-prints, of small cohort studies on COVID-19 are increasing exponentially [39], it becomes clear that further systematic research is required to clarify the potential links between adverse COVID-19 outcomes and factors such as circulating androgens in both male and female COVID-19 patients.

### Hyper-inflammation

It is noteworthy that the severity of COVID-19 in certain cases seems to also relate to predisposition for developing a cytokine storm syndrome with excessive release of pro-inflammatory cytokines (e.g., tumor necrosis factor-α, chemokines, and interleukins such as IL-6, IL-7, IL-8, IL-2, and IL-1β) at the infected lung tissue [40, 41]. Indeed, available data suggest that this syndrome may induce self-sustaining hyper-inflammation reactions in a sub-group of patients with severe COVID-19, leading to respiratory and multi-organ failure [40]. Of note, hyper-cytokinemia and activated pro-inflammatory pathways are also considered to promote the pathogenesis and cardio-metabolic complications of PCOS, particularly when combined with central obesity [19, 23]. It is now-well established that women with PCOS and obesity exhibit marked adipose tissue dysfunction and dysregulated adipokine/cytokine secretion (e.g., increased release of leptin, tumor necrosis factor-α, and IL-6) which result in a chronic pro-inflammatory state [19, 23]. Furthermore, compared to healthy controls, women with PCOS seem to frequently have polymorphisms in genes encoding pro-inflammatory cytokines, such as tumor necrosis factor-α and IL-6 [42, 43]. Both the latter cytokines have also been reported to induce theca cell steroidogenesis by upregulating enzymes involved in ovarian androgen synthesis [19], while ACE-2 is widely expressed in the ovary [44]. Of note, the androgen receptor is also known to play a role in adaptive and innate immunity, particularly in macrophage and neutrophil recruitment, which are closely linked with COVID-19 in the lungs [40]. Therefore, it is plausible that chronic inflammation may constitute another aspect of the PCOS pathophysiology which may be of relevance to COVID-19-related hyper-inflammation (Fig. 1), as has also been suggested for diabetes [45].

### Ethnic background

Recent mortality data strongly indicate that Black, Asian, and Minority Ethnic (BAME) groups are at increased risk of death from COVID-19 even after adjusting for multiple other covariates [9, 10, 46, 47]. Indeed, an analysis of COVID-19-related deaths by ethnic group in England and Wales from the UK Office for National Statistics has shown that the risk of COVID-19-related death is significantly higher in adults of Black, Bangladeshi and Pakistani, Indian, and mixed ethnicities compared to that of adults of White ethnicity [48]. Although less prominent, this risk remained significantly increased after accounting for age and other socio-demographic characteristics, as well as measures of self-reported disability and health [48]. This further indicates that the difference in COVID-19 mortality between ethnic groups is only partly explained by socio-economic disadvantage and other related parameters [48]. Notably, it has also been documented that 63% of a series of 106 healthcare workers who have died from COVID-19 were of BAME background [49]. Multiple factors are considered to contribute to the noted higher risk of BAME groups for COVID-19 severity and mortality, such as higher rates of the aforementioned cardio-metabolic comorbidities and inequalities due to lower socio-economic status, as well as behavioral and cultural differences (e.g., inter-generational cohabitation with less social distancing at households) [47, 50]. The potential role of various genetic factors in this association has also been hypothesized, including ethnicity-related differences in genetic variations of the androgen receptor [32, 47]. In this context, it should be highlighted that substantial ethnic variation has also been documented regarding the diagnosis and presentation of PCOS [27, 51, 52]. Again significant overlap exists here with the noted ethnic predisposition for adverse COVID-19 outcomes, with increasing data showing that particularly women of African American, Hispanic, Middle Eastern, and South Asian origin exhibit more frequent and worse cardio-metabolic features of PCOS [27, 51, 52]. Moreover, certain IL-6 polymorphisms appear to influence PCOS susceptibility in Caucasians, while certain tumor necrosis factor-α polymorphisms might influence PCOS susceptibility in Asians [43]. As such, clinicians should not overlook the fact that BAME background may also be added to the overlapping factors which promote both an unfavorable cardio-metabolic profile in women with PCOS and severe COVID-19 (Fig. 1).

### Low vitamin D levels

Since COVID-19 has been declared a pandemic, potential links between low vitamin D levels and COVID-19 severity have been hypothesized [53–61]. This was triggered by the fact that this pandemic evolved rapidly in several Northern hemisphere countries (e.g., Italy, Spain, France, and UK) during and shortly after winter when sun exposure and vitamin D levels are typically lowest, while COVID-19 cases remained relatively low in the Southern hemisphere during the end of summer [55–58]. Moreover, existing evidence indicates that vitamin D deficiency can contribute to acute respiratory distress syndrome (ARDS), while COVID-19-related mortality increases with both older age and cardio-metabolic comorbidity which also exhibit positive associations with lower vitamin D levels [55–58]. Interestingly, a recent cross-sectional analysis of data on vitamin D levels and COVID-19 morbidity/mortality for 20 European countries showed negative correlations between mean vitamin D levels and both the number of COVID-19 cases and COVID-19 mortality in each country [60]. Of note, particularly low vitamin D levels were noted in the aging populations of Italy and Spain, which were among the main epicenters of the COVID-19 pandemic in Europe [60]. However, such cross-sectional data have obvious limitations, while a recent UK Biobank-based study (data on UK Biobank participants, aged 37–73 years, of whom 449 had confirmed COVID-19) showed that vitamin D was associated with COVID-19 univariably, but not after adjustment for confounders [62]. Hence, this study concluded that these findings do not support a potential link between COVID-19 and vitamin D levels [62]. Nevertheless, vitamin D is a well-known pleiotropic hormone which modulates the adaptive and innate immune responses, and can regulate the IL-6 activity and suppress the pro-inflammatory cytokine response of macrophages and respiratory epithelial cells to various viruses [53, 54, 57, 58]. Thus, the potential role of low vitamin D levels in COVID-19 severity and the development of a related cytokine storm syndrome merits further well-designed research [53, 54, 57, 58]. Notably, growing data support an inverse association between vitamin D and the severity of multiple PCOS manifestations, including hyperandrogenism, infertility, insulin resistance, and cardio-metabolic disease [63, 64]. Furthermore, meta-analysis data indicate that vitamin D supplementation in women with PCOS might significantly decrease total testosterone and C-reactive protein circulating levels, while it can increase the level of total antioxidant capacity [65, 66]. Taken together, these data suggest that women with PCOS may be at potentially higher risk for severe COVID-19 also due to low vitamin D levels (Fig. 1), which may be further aggravated by reduced sun exposure due to COVID-19-related quarantine measures.

## Potential COVID-19-related implications on aspects of PCOS treatment

In addition to the aforementioned frequent conditions/factors which are common in PCOS and may increase the risk for severe COVID-19 in this female patient population, it is also important to highlight certain points which have been the subject of discussion in the recent scientific literature in relationship to COVID-19 and which also relate to aspects of PCOS treatment (Table 1).

**Table 1:** Potential COVID-19-related implications on aspects of polycystic ovary syndrome (PCOS) management (in women with PCOS who are diagnosed with COVID-19, a relevant risk assessment should be performed by the treating physician and any existing treatment should be promptly re-evaluated and optimized, as clinically indicated)

A) For women with PCOS on off-label metformin treatment: it is advisable to consider discontinuing metformin when diagnosed with COVID-19, particularly when symptoms of severe COVID-19 are present and if they become unstable, as also recommended for patients with diabetes	
B) For women with PCOS and diabetes:	
- Should consider discontinuing sodium-glucose co-transporter-2 inhibitors (SGLT2i) when COVID-19 is diagnosed, particularly when symptoms of severe COVID-19 are present	
- Should continue and optimize insulin therapy, as clinically indicated	
- Can continue the use of dipeptidyl peptidase-4 (DPP4) inhibitors where clinically indicated (the dose of certain DPP4 inhibitors may need adjustment if renal function is affected in severe COVID-19)	
- Should consider avoiding/optimizing the use of sulfonylureas when COVID-19 is diagnosed, particularly when symptoms of severe COVID-19 are present, due to the risk of hypoglycemia	
C) Women with PCOS and known or suspected insulin resistance (known type 2 diabetes or prediabetes) who receive glucocorticoid treatment for COVID-19 may exhibit overt glucocorticoid-induced glucose/metabolic dysregulation	
D) Women with PCOS and hypertension may continue treatment with common antihypertensive drugs [angiotensin converting enzyme (ACE) inhibitors, angiotensin-receptor blockers (ARBs), thiazide diuretics, calcium-channel blockers and beta-blockers], as indicated, since available data so far indicate that these appear to not substantially increase the risk for testing COVID-19 positive and for severe COVID-19	
E) Women with PCOS and obstructive sleep apnea (OSA) may continue home use of continuous positive airway pressure (CPAP) therapy, but, particularly in cases of home self-isolation due suspected or confirmed COVID-19, these patients should consider either taking strict quarantine measures from other household members (e.g., use of separate bedrooms and bathrooms where feasible) or discontinuing CPAP therapy for a short period, due to potential risk of SARS-CoV-2 aerosolized transmission from the CPAP use. During any such temporary CPAP therapy discontinuation, sedating medications and alcohol should be avoided, while other measures to reduce OSA can also be considered, such as positional therapy during sleep, nasal congestion treatment, and dental appliance use where possible	

### Metformin treatment

In several countries, metformin is frequently prescribed off-label for the management of women with PCOS and increased BMI, even without co-existing T2DM, since it can improve both reproductive and metabolic outcomes in these patients [19, 20]. Historically, it is noteworthy that metformin was essentially rediscovered in the 1940s during the search for anti-malarial agents, when it was shown useful to treat influenza and also to lower blood glucose as a side effect [67]. Giver the former use, it is not surprising that metformin has been considered among the drugs which could be repurposed against COVID-19, with potential antiviral effects through activation of the AMP-activated protein kinase (AMPK) pathway that would cause changes in the ACE-2 receptor and block the entry of SARS-CoV-2 into host cells [68]. However, metformin use can promote lactic acidosis in the context of marked dehydration and renal impairment in severe COVID-19; hence, women with PCOS under metformin treatment is advisable to consider stopping this agent when symptomatic with COVID-19, particularly if they become unstable, as also recommended for patients with diabetes [69–71].

### Other glucose lowering medications

As with metformin, women with PCOS and diabetes should follow the recent practical recommendations and guidelines issued for antidiabetic medications in view of COVID-19 [69, 70]. Accordingly, due to the related risk of diabetic ketoacidosis and dehydration from sodium-glucose co-transporter-2 inhibitors (SGLT2i), discontinuing these agents should be considered when COVID-19 is diagnosed, particularly in symptomatic patients [69–71]. Conversely, insulin therapy should be continued and optimized, while it remains the treatment of choice for severely ill patients with diabetes and COVID-19 [69–71]. Furthermore, the use of dipeptidyl peptidase-4 (DPP4) inhibitors seems to be mostly well-tolerated and can also be continued where indicated (the dose of certain DPP4 inhibitors may need to be adjusted depending on whether the renal function is affected in severe COVID-19) [69–71]. Interestingly, the ubiquitously expressed membrane-associated human DPP4 appears to be a functional receptor for the Middle East respiratory syndrome coronavirus (MERS-CoV), but not for SARS-CoV-2 [71]. Finally, due to the related increased risk of hypoglycemia, avoiding/optimizing the use of sulfonylureas should also be considered in patients with severe COVID-19 [71].

In the context of glucose control in women with PCOS and IGT or T2DM, a note should also be made regarding potential glucocorticoid treatment which is administered in certain hospitalized COVID-19 patients [72]. Indeed, such glucocorticoid treatment for COVID-19 may aggravate the glucose and metabolic homeostasis [17, 73], particularly in women with PCOS who may have significant underlying (and often undiagnosed) insulin resistance [19, 23, 24]. As such, in these cases treating physicians should be on high alert regarding the potential glucocorticoid-induced glucose/metabolic dysregulation and mitigate this as indicated.

### Antihypertensive medications

Given the role of ACE-2 in facilitating the entry of SARS-CoV-2 into host cells, the use of ACE inhibitors for hypertension treatment in the setting of COVID-19 has been another point of recent debate [74–78]. Therefore, as hypertension is also common in women with PCOS [19, 20, 24], it is important to note that a recent study with relevant data on five common antihypertensive drug classes, namely ACE inhibitors, angiotensin-receptor blockers (ARBs), thiazide diuretics, calcium-channel blockers, and beta-blockers, showed no substantial increase in the risk for testing COVID-19 positive and for severe COVID-19 [74]. Overall, increasing data so far show no evidence that ACE inhibitors or ARBs increase the risk of COVID-19, supporting current guidelines recommending to continue the use of these drugs as indicated despite this pandemic [74–78]. Of note, additional data from two cohorts of men and women with heart failure showed that treatment with ACE inhibitors or ARBs was not associated with higher plasma ACE-2 [79].

### Home use of continuous positive airway pressure (CPAP) therapy

OSA exhibits high prevalence among women with PCOS and obesity, attributed at least partly to hyperandrogenism and central adiposity, and further aggravates the adverse cardio-metabolic profile of these patients [19, 25, 80, 81]. As OSA may cause various degrees of reduced arterial oxygen saturation and additional cardio-metabolic complications, routine CPAP home use is often indicated in women with PCOS and OSA [19, 25, 80, 81]. However, because CPAP is regarded as a high-risk aerosol-generating procedure which may increase the risk of SARS-CoV-2 transmission to other household members, routine CPAP therapy at home has been the subject of recent debate [82–84]. Particularly for any OSA patient with suspected or confirmed COVID-19 who is self-isolating at home, it is reasonable to consider discontinuing CPAP therapy for a short period due to the potential risk of SARS-CoV-2 aerosolized transmission from the CPAP use to other household members [82, 85]. Alternatively, strict distancing measures within the household (e.g., changing bedrooms and using different bathrooms where feasible) should be taken in order to protect household contacts [82, 85]. During any period of necessary CPAP discontinuation, OSA patients should be informed of the related risk/consequences and avoid sedating medications and alcohol use [82]. In addition, during such periods of temporary CPAP discontinuation, other practical measures to reduce OSA-related symptomatology can be also considered, such as positional therapy during sleep (head elevated at least 30° or side sleeping), nasal congestion treatment, and dental appliance use where possible [82].

## Practical recommendations for the management of PCOS during the COVID-19 pandemic

Given the aforementioned overlap between risk factors for severe COVID-19 and common PCOS features, and the implications that a COVID-19 diagnosis may have on various aspects of PCOS care, it is crucial for the clinical practice to recognize that closer monitoring and revised management plans may be required for this female patient population during this pandemic. As such, clinicians treating women with PCOS, irrespective of their specialty, should assess the risk profile of these patients in relationship to COVID-19. Depending on the outcome of this risk assessment regarding coexisting comorbidities which may predispose to severe COVID-19, relevant advice should be provided about issues such as shielding and self-isolation, as well as practical recommendations for treatment changes/optimization like those outlined in Table 1. For example, women with PCOS and T2DM and/or hypertension should be informed of the potential higher risk for severe COVID-19, and if diagnosed with COVID-19, their treatment should be promptly re-evaluated and optimized, as indicated (Table 1). Further clinical input may also be required in order to apply a more individualized care plan for women with PCOS who may have additional management issues during this pandemic. Indeed, the rates of certain pregnancy complications (e.g., preterm birth, cesarean delivery, miscarriage, gestational diabetes, and pregnancy-induced hypertension and preeclampsia) are higher in this female patient population [86–89]. Therefore, taking into account that based on recent meta-analysis data the so far reported rates of preterm birth and cesarean delivery are higher in pregnant women with COVID-19 (although their clinical picture may not differ from the non-pregnant population) [90], closer antenatal and perinatal monitoring is required in pregnant women with PCOS, particularly when hypertension and/or diabetes are also present. Furthermore, women with PCOS also exhibit higher risk for mental health problems (e.g., anxiety and depression) [19, 20]. Such problems may be triggered and/or exacerbated in the setting of the COVID-19 pandemic (e.g., due to issues relating to prolonged shielding, isolation, job insecurity, and fear of ill health) [91]. Thus, clinicians should recognize this risk in this vulnerable female patient population and offer appropriate mental health support for prevention and treatment as needed. Moreover, it should be mentioned that COVID-19 appears to predispose to thrombosis [92, 93], and so a COVID-19 diagnosis should also prompt heightened vigilance for potential thromboembolic complications in women with PCOS who may already be at increased thrombotic risk, particularly when additional pro-thrombotic factors are also present (e.g., obesity, treatment with combined oral contraceptives) [94]. Finally, as COVID-19 is a new disease which is now being studied systematically, clinicians should keep in mind that there may be unpredictable, longer term consequences following a SARS-CoV-2 infection which are yet unknown (e.g., long-term dysregulation of endocrine functions and metabolic homeostasis) [95]. Therefore, follow-up of women with PCOS who recover from COVID-19 should also be implemented in clinical practice in order to promptly diagnose and treat any such potential long-term complications of COVID-19 [95].

## Conclusions

At a first glance, women with PCOS appear to belong to an age and sex group which is considered as less affected by COVID-19. However, it should be highlighted that within this common female patient population there is high prevalence of numerous conditions/factors which may significantly increase the risk for worse COVID-19-related outcomes. This strong overlap should not be overlooked in clinical practice, particularly since women with PCOS often receive fragmented care from multiple healthcare services. In parallel, informing women with PCOS regarding the potential risks from COVID-19 and how this may affect their management is also necessary. Overall, despite the immense challenges posed by the COVID-19 outbreak in affected countries, attention should be directed to maintain a high standard of care for complex patients, such as many women with PCOS, and provide relevant practical recommendations for optimal management in the setting of this fast moving pandemic.

## Data Availability

Not applicable.

## Abbreviations

ACE-2: Angiotensin-converting enzyme 2
AMPK: AMP-activated protein kinase
ARBs: Angiotensin-receptor blockers
ARDS: Acute respiratory distress syndrome
BAME: Black, Asian, and Minority Ethnic
BMI: Body mass index
CPAP: Continuous positive airway pressure
DHEAS: Dehydroepiandrosterone sulphate
DPP4: Dipeptidyl peptidase-4
IGT: Impaired glucose tolerance
IL: Interleukin
MERS-CoV: Middle East respiratory syndrome coronavirus
NAFLD: Non-alcoholic fatty liver disease
OSA: Obstructive sleep apnea
PCOS: Polycystic ovary syndrome
SARS-CoV-2: Severe acute respiratory syndrome coronavirus-2
SGLT2i: Sodium-glucose co-transporter-2 inhibitors
SHBG: Sex hormone binding globulin
T2DM: Type 2 diabetes mellitus
